# Supporting the “forgotten” ventricle: The evolution of percutaneous RVADs

**DOI:** 10.3389/fcvm.2022.1008499

**Published:** 2023-01-04

**Authors:** Les James, Deane E. Smith

**Affiliations:** Department of Cardiothoracic Surgery, NYU Grossman School of Medicine, New York, NY, United States

**Keywords:** heart failure, right ventricular assist device (RVAD), right ventricular (RV) failure, ProtekDuo, percutaneous right ventricular assist device

## Abstract

Right heart failure (RHF) can occur as the result of an acute or chronic disease process and is a challenging clinical condition for surgeons and interventionalists to treat. RHF occurs in approximately 0.1% of patients after cardiac surgery, in 2–3% of patients following heart transplantation, and in up to 42% of patients after LVAD implantation. Regardless of the cause, RHF portends high morbidity and mortality and is associated with longer hospital stays and higher healthcare costs. The mainstays of traditional therapy for severe RHF have included pharmacological support, such as inotropes and vasopressors, and surgical right ventricular (RV) assist devices. However, in recent years catheter-based mechanical circulatory support (MCS) strategies have offered novel solutions for addressing RHF without the morbidity of open surgery. This manuscript will review the pathophysiology of RHF, including the molecular underpinnings, gross structural mechanisms, and hemodynamic consequences. The evolution of techniques for supporting the right ventricle will be explored, with a focus on various institutional experiences with percutaneous ventricular assist devices.

## Introduction

Right heart failure (RHF) is characterized by systemic congestion as a result of reduced right ventricular (RV) forward flow. RHF is a challenging clinical condition that can occur as the end result of chronic disease, including decompensated biventricular heart failure due to cardiomyopathy or left-sided ischemic and valvular diseases, cor pulmonale, and pulmonary hypertension (pHTN). Acute processes such as myocardial infarction, postcardiotomy cardiogenic shock, massive pulmonary embolism, heart transplantation, and left ventricular assist device (LVAD) implantation can also lead to acute RHF (1–4). Clinically significant RHF occurs in approximately 0.1% of patients after cardiac surgery, in 2–3% of patients following heart transplantation, and in 9–44% of patients after LVAD implantation, depending on diagnostic criteria used (5–8). It must be emphasized that the lack of diagnostic clarity regarding what constitutes RHF post-LVAD significantly limits strategies to predict or prevent its incidence. Regardless of the cause, RHF portends high morbidity and mortality and is associated with longer hospital stays and higher healthcare costs (3, 9, 10).

Standard therapy for severe RHF consists of pharmacological support to improve myocardial contractility, including inotropes and vasopressors, as well as volume unloading and the application of pulmonary vasodilators (e.g., prostaglandin and nitric oxide) (11). If maximal medical therapy fails, mechanical circulatory support (MCS) is necessary to restore blood flow to the pulmonary circulation and left ventricle (LV) (11). MCS with a right ventricular assist device (RVAD) is an established treatment strategy for RHF. The right atrium (RA) and pulmonary artery (PA) are cannulated, typically via median sternotomy, to permit temporary right ventricle (RV) support. However, surgically implanted RVADs (sRVAD) come with significant limitations, including the need to return to the operating room for re-thoracotomy to remove the device and limited capacity for mobilization of patients on support, with all the associated complications therein. Several studies have reported high morbidity and mortality with the repeat sternotomies required of sRVAD (12–14). Catheter-based MCS solutions have become an attractive option for supporting RHF refractory to medical management. Recent studies have suggested that minimally invasive support strategies can be beneficial, although the literature regarding the efficacy of these devices in the management of acute RHF has been limited to case reports and case series (3, 15–21).

This manuscript will review the pathophysiology of RHF and the evolution of mechanical circulatory devices for supporting the RV. The current literature will be surveyed, with a specific emphasis on various institutional experiences with percutaneous RVADs.

## Pathophysiology of right heart failure in cardiac surgery

The RV has long been regarded as the “forgotten” cardiac chamber, as historically the emphasis in both cardiology and cardiac surgery has been placed on the LV and its ejection fraction. Consequently, RHF has historically been a poorly understood pathology, relegated to an afterthought despite substantial advances in the understanding and management of left heart failure. Owing to the complex nature of RHF, the International Right Heart Foundation was convened with the mandate of devising a common language to describe right RHF. The group defined RHF as “a clinical syndrome due to an alteration of structure and/or function of the right heart circulatory system that leads to suboptimal delivery of blood flow (high or low) to the pulmonary circulation and/or elevated venous pressures—at rest or with exercise” (22). This broad definition encompasses a spectrum of disorders, including those that always involve the RV to those that are unrelated to the structure and function of the RV (e.g., pretricuspid lesion). It is well established that RV function is a strong predictor of mortality, not only in heart disease but also in pHTN, congenital heart disease, and cardiothoracic surgery (23). RV function serves as a key component of risk stratification for patients undergoing surgery for coronary artery disease, congenital heart disease, and heart transplantation, as well as for patients requiring mechanical assist devices and patients experiencing postoperative hemodynamic instability (5).

To understand the pathophysiology of RHF, it is necessary to begin with a brief overview of some of the molecular mechanisms that may contribute to remodeling of the RV. Macro- and micro-vascular ischemia have both been implicated in the evolution of RV failure. Under physiologic conditions, the RV is perfused during both systole and diastole by the right coronary artery (RCA). In conditions of increased RV afterload there is systolic flow impediment and the myocardium of the RV only receives blood flow during diastole, making adequate perfusion dependent on lower heart rates and longer filling times. Increases in the RV end-diastolic pressure (RVEDP) and RV hypertrophy decrease coronary perfusion pressure and limit flow to the RV myocardium (24, 25). This results coronary remodeling, as demonstrated by the inflammation and smooth muscle hyperplasia seen in the thickened epicardial arteries of patients with pHTN (26). The role of microvascular dysfunction in RV failure is less well understood compared to the large epicardial arterial changes. There is evidence from animal models of pHTN that suggests failure of RV adaptation is associated with a lack of adequate microvascular angiogenesis, as demonstrated by decreased vascular endothelial growth factor mRNA and protein expression (27–29). It is difficult to determine whether therapies to directly modify RV angiogenesis are beneficial, as most medications associated with increased RV vascularization or increased expression of pro-angiogenic mediators also reduce the afterload of the pulmonary vasculature (24). Sex steroids and metabolic derangements, including mitochondrial dysfunction, abnormal fatty acid metabolism, and insulin resistance, also have a complex role underlying RHF. While a detailed discussion of these mechanisms is beyond the scope of this review, we would point the reader to an excellent paper by Agrawal and colleagues for a complete overview (24).

The physical properties of the RV also play an important role in the pathophysiology of RHF. Triangular in shape and crescent on cross-section, the RV has a lower ejection fraction compared to the LV, with a normal range of 40–45%. To generate the same stroke volume and cardiac output, the RV maintains a higher end-diastolic volume (4). The function of the RV is determined by the orientation of its fibers, as described by the helical ventricular myocardial band model (1). The transverse fibers that predominate in the thin free wall of the RV undergo longitudinal, sequential contraction, narrowing the cavity, and accounting for 20–30% of the RV ejection fraction. Conversely, the interventricular septum has helical fibers that twist and shorten the longitudinal axis of the RV, accounting for approximately 80% of the RV ejection fraction. Both normal septal position and twisting motion are essential for adequate RV function. The RV is afterload sensitive and as pulmonary vascular resistance or other sources of RV afterload increase, stroke volume decreases in a linear fashion (4). Dilation and distension of the RV can distort the helical fiber orientation of the septum, resulting in less efficient ejection. Furthermore, pathophysiologic states that result in septal dysfunction, such as ischemic, non-ischemic, and valvular cardiomyopathies, cause paradoxical septal motion (PSM). PSM is the systolic movement of the interventricular septum toward the RV, which may lead to wall motion dysfunction that limits the contractile capacity of the RV. Reynolds et al. found that severe intraoperative septal dysfunction resulting in PSM developed in almost 50% of 3,300 adult cardiac surgery patients whose septum had been normal preoperatively (30). Aortic and mitral valve surgery, as well as longer cardiopulmonary bypass (CPB) times, were independently associated with PSM. Additionally, coronary artery bypass grafting (CABG) was less likely to cause PSM than non-CABG surgery, and off-pump CABG caused less PSM compared to on-pump CABG (30). Although temporary septal dysfunction has been understood as a usual occurrence following cardioplegic arrest of the heart, enduring damage may not be immediately apparent. As transthoracic echocardiography (TTE) can only visualize the RV free wall, acute RHF is only suspected when the free wall has become significantly dilated. Consequently, the diagnosis of RHF and its underlying etiology may be delayed. This highlights the importance of adequate myocardial protection to ensure preserved septal function.

There are four primary mechanisms which underlie the development of acute RHF: contractile failure secondary to myocardial ischemia or inflammation caused by myocarditis, volume overload as a result of right-sided valvular insufficiency, volume overload caused by increased venous return or displacement of the interventricular septum toward the LV after placement of a LVAD, and pressure overload resulting from decompensated left-sided heart failure, worsening pHTN, or acute pulmonary embolism (Table 1) (5, 31). Postcardiotomy RHF is often precipitated by an element of ischemia and myocardial depression after CPB (5). In patients requiring LVAD support, unloading of the LV leads to alterations in the size and shape of the RV, which may lead to acute RHF. After cardiac transplantation, donor heart ischemia, and preexisting pulmonary vascular disease increase the risk of postoperative RHF.

**TABLE 1:** Common causes of right ventricular failure in cardiac surgery.

Mechanism of postoperative RV failure	Specific etiologies
Preexisting RV dysfunction	● Preoperative RV dysfunction associated with pulmonary hypertension or congenital, valvular, or coronary disease
RV myocardial infarction	● Coronary embolism (air, thrombus), thrombotic occlusion, graft dysfunction
Postsurgical myocardial dysfunction	● Suboptimal myocardial protection, long CPB time
Postoperative pulmonary hypertension	● Preexisting pulmonary hypertension ● Ischemia-reperfusion injury ● Pulmonary embolism ● LV failure ● Excessive blood transfusions
Dynamic obstruction of the RVOT	● Volume depletion ● High dose of inotropes
Excessive volume loading of the RV	● Excessive transfusions or volume infusion ● Severe tricuspid regurgitation
Acute unloading of the LV	● Following initiation of LVAD support
Cardiac transplantation	● Pulmonary hypertension ● Prolonged ischemic time ● Acute rejection ● Obstruction at the PA anastomosis
Pericardial constriction	● Postcardiotomy syndrome

CPB, cardiopulmonary bypass; LV, left ventricular; LVAD, left ventricular assist device; PA, pulmonary artery; RV, right ventricular; RVOT, right ventricular outflow tract. Adapted with permission from Haddad et al. (5).

Following LVAD insertion, the incidence of acute RHF ranges from 9 to 42% depending on diagnostic criteria, study population, and institution, and is associated with a high mortality (8, 9, 32–35). Definitions of acute RHF post-LVAD vary widely, and have included the need for RVAD, the use of inhaled pulmonary vasodilators, or the prolonged use or delayed reinstitution of inotropes. The Interagency Registry for Mechanically Assisted Circulatory Support (INTERMACS) defines RHF as (1) the need for an RVAD or (2) requiring inhaled nitric oxide or inotropic therapy for greater than 1 week any time after LVAD implantation in the presence of symptoms and signs of persistent RV dysfunction, such as central venous pressure > 18 mmHg with a cardiac index < 2.3 L/min/m^2^ in the absence of elevated left atrial or pulmonary capillary wedge pressure (>18 mmHg), cardiac tamponade, ventricular arrhythmias, or pneumothorax (36). A lack of diagnostic clarity compounds the challenge of managing acute RHF post-LVAD. Importantly, RHF will arise in patients with an LVAD even if the pulmonary vascular resistance was initially low, as the resistance increases progressively in the setting of lung endothelial injury secondary to blood and platelet transfusions (1). The RV free wall, with its transverse fibers, cannot maintain normal RV output, leading to decreased LV venous return, and diminished LV cavity size. As a result, the interventricular septum bulges toward the LV, changing the orientation of the helical fibers of the septum and impairing its twisting motion. This alteration in septal position is visible on TTE and usually requires immediate attention to prevent suction events. Reducing LVAD speed and increasing pharmacologic support to the RV are the initial steps in management. Ochiai et al. reviewed preoperative data for patients who underwent LVAD implantation and found that the most significant risk factors for RVAD use after LVAD were the need for MCS prior to LVAD, female gender, and a non-ischemic etiology of RHF (32). In this study, important hemodynamic variables that predicted RVAD use after LVAD were low mean PA pressure and low RV stroke work index. A low mean PA pressure reflects the inability of the failing RV to generate high or even normal PA pressures.

Multiple hemodynamic indices are used to characterize the physiology of the RV, including RV afterload, right atrial pressure (RAP), the RAP-to-pulmonary artery wedge pressure (RAP/PAWP) ratio, RV stroke work index, the pulmonary artery pulsatility index (PAPi), and tricuspid annular plane systolic excursion (TAPSE) (37, 38). While all correlate with RHF, their sensitivity and specificity for predicting outcomes can be quite variable. The RAP/PAWP ratio is a relatively poor predictor in patients with RHF post-LVAD. The PAPi is a recent addition to the hemodynamic measures of RV function and in a prospective study of clinical indices of RHF, Aslman and colleagues identified it as an excellent predictor of underlying RV myofilament contractility (37, 39). TAPSE is a routinely obtained echocardiographic parameter of global RV function that measures the longitudinal deformation of the RV free wall; it is closely correlated with RV ejection fraction (40, 41). TAPSE has gained traction as a more reliable RHF risk-stratification and prognostic tool. In a recent study by Read and colleagues, the authors retrospectively analyzed patients who had undergone continuous-flow LVAD implantation and who had vasodilator testing with nitroprusside during right heart catheterization prior to implant (42). In multivariable analysis the study found that peak stroke volume index (SVI) was significantly associated with early RHF, with a 16% increase in the risk of early RHF per 1 mL/m^2^ decrease in SVI. This suggests that assessing RV reserve may be useful in predicting which patients are at risk for RHF post-LVAD.

Given that patients who receive LVADs and develop acute RHF have poor outcomes, there has been significant interest in developing clinical models to better predict which patients are at the highest risk (9, 43–47). These clinical models include patients’ demographics and their current medications, hemodynamic profile prior to LVAD implantation, and laboratory markers of organ damage. A study by Kalogeropoulos et al. evaluated 6 clinical RHF prediction models in a continuous-flow LVAD cohort and found that these models have limited applicability, especially in the absence of quantitative pre-operative imaging data (48).

Despite advances in the perioperative management of patients undergoing heart transplantation, acute RHF accounts for a significant number of early complications and early deaths (49). Multiple factors contribute to the development of acute RHF following heart transplant, including preexisting or acquired pHTN, marginal organ preservation and long ischemic times, mechanical obstruction at the level of the PA anastomosis, significant donor-recipient mismatch with a smaller donor heart, and acute allograft rejection (5, 50). RHF following transplantation may be revealed by high RA pressures, which has traditionally been attributed to the persistently high PVR that exists in the recipients. However, some degree of septal injury in the donor heart sustained during prolonged ischemia may diminish the twisting capacity of the septum, limiting its ability to generate adequate pulmonary blood flow in patients with an elevated PVR (1). Novel reperfusion methods, such as leukocyte removal filters, may prevent this septal damage, an injury that never existed in the healthy donor heart (51). The resultant normal septal performance will also reduce the prolonged use of inotropes or pulmonary vasodilators.

## The evolution of percutaneous right ventricular assist devices

In cases where acute RHF results from a mechanical insult in the perioperative setting, such as graft occlusion following CABG, compression of the PA, or stricture at the PA anastomosis (e.g., heart transplantation), surgical or procedural interventions to address the underlying cause may be sufficient to improve the function of the failing ventricle. Pharmacotherapy for acute RHF focuses on managing volume and preload, improving myocardial contractility, and reducing RV afterload (52). Volume management strategies include early, aggressive high-dose diuretics and for patients who fail to respond, early initiation of renal replacement therapy. Vasoactive medications also play an important role in the management of acute RHF, although there are few clinical trials to guide their selection. A PA catheter is often helpful to trend biventricular filling pressures and cardiac output. Vasodilators, such as nitroglycerin and sodium nitroprusside, decrease both PVR and systemic vascular resistance and result in improved RV and LV stroke volume. Inotropes can augment RV contractility and simultaneously reduce RV end-diastolic volume and pressure. Milrinone and dobutamine act as both inotropes and vasodilators and have similar hemodynamic efficacy, although must be used with caution as they can precipitate or worsen hypotension in acute RHF. In patients with acute RHF and concomitant significant hypotension, dopamine, norepinephrine, and epinephrine are the vasopressors of choice to maintain perfusion. Their use must be carefully weighed against the risks of increasing ventricular afterload. Ultimately, most cases of refractory RHF will require some form of MCS. Historically, surgically implanted pulsatile PA balloon pumps with valves located within the inflow and outflow cannulae were used to support the failing RV (31). In the early 1990s, rotary-flow RVADs demonstrated hemodynamic superiority and better clinical outcomes compared with PA balloon counterpulsation pumps for acute RVF; thus the use of PA balloon pumps was abandoned (6, 31).

Most MCS options for supporting the right heart include cannulation of the RA and the PA to deliver flow into and out of a continuous flow pump, respectively. The pressure gradient between the RA pressure (preload) and the PA pressure (afterload) is referred to as the pressure head and varies throughout the cardiac cycle. In patients with severe RHF resulting from acute ischemia or impaired cardiac contractility, the pressure head is often small and for fixed rotations per minute, device flow will be high. Conversely, for patients with RHF caused by severe pHTN, the pressure head may be large, and for the same rotations per minute setting, device flow will be low (31).

There are several relatively new minimally invasive RVAD strategies that enable intervention on the failing RV without the morbidity of open surgery for device removal. Temporary RVAD configurations include venoarterial extracorporeal membrane oxygenation (VA-ECMO), surgically implanted grafts combined with a centrifugal-flow pump, and percutaneous RVADs (pRVAD), including the Impella RP (Abiomed Inc., Danvers, MA) and the ProtekDuo (TandemLife, Pittsburgh, PA) cannula. There are currently no U.S. Food and Drug Administration (FDA)-approved long-term implantable devices designed to support the RV. Most reports of long-term RV support have been limited to off-label applications of devices designed and approved for the treatment of advanced LV failure, such as the HeartWare Ventricular Assist Device (HVAD; Heart-Ware, Framingham, MA) and the Heartmate 3 (St. Jude Medical, St. Paul, MN) (53–60). Other solutions have relied on adapting biventricular support devices for long-term RV support. The Berlin Heart EXCOR (Berlin Heart GmbH) is a paracorporeal assist device that can be used for long-term bridging therapy of the RV, although it is not currently FDA approved and is therefore not available for RVAD use in the United States (61).

Temporary RVAD configurations can be categorized according to their mechanism of action as either direct or indirect RV bypass systems (31). The Impella RP and other pRVAD configurations displace blood from the RA to the PA, directly bypassing the RV. In patients with isolated RVF, either of these devices will directly reduce RA pressure, increase mean PA pressure, and increase LV preload. In the presence of preserved LV function, native cardiac output will increase, LV filling pressures will increase or remain the same, and LV afterload will be unchanged. Conversely, VA-ECMO is an indirect RV bypass, as it displaces blood pulled from the RA, oxygenates it, and returns it to the femoral artery. VA-ECMO initially decreases RA and PA pressures and decreases LV preload. LV afterload will increase, and therefore, in the presence of preserved LV function, cardiac output may remain unchanged or decrease. In cases of biventricular failure, it is paramount to consider these effects when initiating mechanical RV support (Table 2) (62–64).

**TABLE 2:** Temporary mechanical circulatory support devices for acute right ventricular failure.

Device	Description of cannulation	Ventricular support	Cardiac output augmentation	Arterial cannula size and site	Venous cannula size and site	Alternative access sites	Advantages	Disadvantages	Contraindications
VA-ECMO	Centrifugal extracorporeal pump with membrane oxygenator	Biventricular volume and pressure unloading	2–6 L/min	15–22 Fr Femoral artery	18–21 Fr Femoral vein	Axillary artery and internal jugular vein Central cannulation	● Support for biventricular failure ● Hybrid and dynamic configuration ● Relatively inexpensive ● Rapid deployment with cutdown technique or percutaneous insertion	● Infection ● Leg ischemia ● Hemolysis ● Vascular and bleeding complications ● Differential hypoxia for impaired lung function ● Flow competition with LVAD (thrombosis LVAD) ● No specific support for RVF ● High complexity with perfusionist supervision required and specialized ICU monitoring	● Aortic valve insufficiency ● Peripheral arterial disease
Impella RP	Intracorporeal micro-axial flow pump; inserted with the inflow in the IVC and the outflow in the PA	RV volume and pressure unloading	2–4 L/min	N/A	23 Fr Femoral vein	N/A	● Early insertion ● Single venous access site ● Small dimension of the machine support ● Moderate complexity with specialized ICU monitoring	● Hemolysis ● Vascular and bleeding complications ● Worsened tricuspid or pulmonary valve dysfunction (rare) ● No mobilization due to easy dislocation of the device ● No ability to oxygenate blood	● Any contraindication to anticoagulation ● Significant pulmonic or tricuspid stenosis or regurgitation
Paracorporeal RVAD	Extracorporeal centrifugal pump with or without membrane oxygenator; inflow cannula in the RA and outflow in the PA	RV volume unloading	2–4 L/min	N/A	29–31 Fr Internal jugular vein (ProtekDuo cannula)	Central cannulation	● Easy insertion ● Isolated RV support ● Possibility to add an oxygenator in the circuit leading to central oxygenation (physiologic “oxy-RVAD”)	● Perforation of right heart chambers or PA ● Pulmonary insufficiency ● Arrhythmias ● High complexity with specialized ICU monitoring	● Any contraindication to anticoagulation ● Venous stenosis ● Significant pulmonic or tricuspid stenosis or regurgitation

CI, cardiac index; CVP, central venous pressure; IJV, internal jugular vein; IVC, inferior vena cava; LV, left ventricle; LVAD, left ventricular assist device; MAP, mean arterial pressure, PA, pulmonary artery; RA, right atrium; RV, right ventricle; RVAD, right ventricular assist device; RVF, right ventricular failure; VA-ECMO, veno-arterial extracorporeal membrane oxygenation.

It should be noted that it is difficult to generalize about the utility of pRVADs in the management of patients with acute RHF owing to the variations in the definition of RHF from study to study. Similarly, institutions may also have different thresholds for initiating MCS in acute RHF. The majority of studies have been retrospective in nature, are limited to single-center experiences, and include small numbers of patients. This prohibits any definitive conclusions from being drawn about the indications for pRVAD use and the potential benefits this device may confer.

### VA-ECMO

VA-ECMO is a well-established and effective MCS option for acute RVF, as it can be placed rapidly and peripherally using either a cutdown technique or percutaneous insertion. ECMO provides indirect support to the RV by reducing preload, reducing RV wall tension, and delivering oxygenated blood to the coronary circulation while supporting the systemic circulation and end-organ function (4, 65). The most common cannulation configuration is venous drainage from the femoral vein and arterial inflow via the common femoral artery or axillary artery, although other configurations, including central cannulation, are also used. In addition to being quickly deployed, VA-ECMO is also relatively inexpensive. The primary disadvantage to VA-ECMO is that it is not specifically designed for patients with isolated RVF, as systemic arterial inflow bypasses what may be a normal functioning LV or LVAD, leading to increased LV afterload. This increased afterload can lead to LV distension, increased LV wall stress, and increased myocardial oxygen demands (66). High LV end diastolic pressure can result in subendocardial ischemia, further weakening the LV. Strategies to vent the LV during VA-ECMO are therefore critically important. Additionally, flow competition can lead to thrombosis in the LVAD. Peripheral arterial cannulation can result in limb hypoperfusion and ischemia, although this may be mitigated by the use of anterograde distal limb perfusion cannulae and near-infrared monitoring (67). For patients with RV failure who are peripherally cannulated for VA-ECMO, blood is returned to the arterial circulation via the femoral artery and flows up the aorta in a retrograde fashion. Concurrently the native heart pumps against the circuit, resulting in competitive flow within the aorta and creating a mixing cloud. The upper body receives blood from the native cardiac output, while the lower body is perfused via the ECMO. If there is impaired pulmonary oxygenation or RV dysfunction, poorly oxygenated blood perfuses the upper body and coronary arteries, a phenomenon referred to as “Harlequin Syndrome” or “North-South Syndrome.” VA-ECMO is contraindicated in patients with aortic valve insufficiency, as it will worsen LV distention. Lastly, VA-ECMO requires highly complex multidisciplinary management and perfusionist supervision in an ICU setting. In the United States, the use of VA-ECMO has steadily increased, with a proportional decrease in postcardiotomy patients and increase in non-surgical cardiopulmonary failure (4).

### Impella RP

The Impella RP is an intracorporeal axial-flow pRVAD that uses a 22 Fr microaxial continuous-flow pump mounted on an 11 Fr catheter that diverts blood from the RA into the PA. The device is introduced most commonly via a 23 Fr venous peel-away sheath at the right femoral vein over a 0.025 wire under fluoroscopic guidance and sits across the tricuspid valve and RV, with the pump inflow positioned in the inferior vena cava and the pump outflow situated in the PA. The Impella RP can deliver up to 4 L/min of flow and the intended use of the device is 14 days. Unfortunately groin cannulation is easily dislocated, limiting patient mobilization. After removal, the venous access site is closed with manual compression and a purse-string or deep mattress suture. Importantly, the Impella RP cannot be used to oxygenate blood; in the setting of RHF with concomitant hypoxemic or hypercarbic respiratory failure, alternative support strategies should be considered. The Impella RP has been used successfully for RHF in the setting of cardiac surgery and after LVAD placement. The prospective RECOVER RIGHT study investigated the safety and efficacy of the Impella RP in patients with medically refractory RVF (18 patients after LVAD implantation, 12 patients after postcardiotomy, or acute myocardial infarction) (18). Immediately following Impella RP insertion, central venous pressure and cardiac index improved, facilitating weaning of inotropes, and vasopressor support. Patients were supported for a mean duration of 3 days and the most common adverse events were bleeding and hemolysis. No thromboembolic events were observed, and worsened tricuspid or pulmonary valve dysfunction was a rare occurrence. The primary end-point was survival at 30 days or hospital discharge, which compared favorably to a prior prospective study by John et al. of surgical RVAD in a similar patient population (73 vs. 47%) (13, 18).

Following FDA approval of the Impella RP for the treatment of RHF, Continuous Access Protocol (CA) and Post-Approval Study (PAS) assessments were undertaken to monitor the post-market experience and demonstrate the outcome trends of pooled data of all Impella RP clinical studies in patients presenting with acute RHF (17). This 2018 prospective cohort study included 60 patients with RHF refractory to medical treatment who received the Impella RP device, and the study population encompassed 2 cohorts: Cohort A, patients with RHF post-LVAD implantation; and Cohort B, patients with RHF postcardiotomy, heart transplant, or myocardial infarction. As with RECOVER RIGHT, the primary end-point was survival at 30 days or hospital discharge. Patients were a mean age of 59 years old, and within the cohort 84% had a history of congestive heart failure, 44% had valvular disease, and 35% had pre-operative renal dysfunction. Prior to Impella RP implant, patients received an average of 3 inotropes or vasopressors. Patients were supported with the Impella RP for a mean of 4 days, and both cardiac index and central venous pressure improved immediately after the initiation of device support. The overall survival at 30 days or at hospital discharge was 72% (17).

### Percutaneous RVADs

Surgically implanted temporary RVADs require sternotomy, followed by anastomosis of grafts to the RA and PA. The grafts are subsequently tunneled obliquely through the chest wall and intercostal spaces, enabling chest closure and ambulation. This strategy employs a continuous centrifugal-flow pump within a circuit that is capable of incorporating an oxygenator, which is referred to as an oxy-RVAD (68, 69). A variation of this technique is percutaneous venous drainage via the right internal jugular vein or through the common femoral vein, instead of anastomosing a graft to the right atrium. For patients with postcardiotomy RHF that is appreciated intraoperatively, including during LVAD implantation, placement of a temporary RVAD is a practical support strategy due to the obligate sternotomy. Removal of the RVAD can be performed safely at the bedside with trimming and oversewing of the external portion of the PA graft to the level of the intercostal muscles, with no ill effects of leaving the residual graft in place (70).

The ProtekDuo is a dual-lumen cannula percutaneously inserted in the right internal jugular vein that, when connected to a pump, functions as a pRVAD (3). One lumen serves as an inflow cannula and encompasses a series of inflow vents positioned across the superior vena cava into the RA, receiving venous drainage from both the upper and lower body. The second lumen has a multi-fenestrated distal tip to deliver blood into the main PA, bypassing the RV. The cannula is placed under transesophageal echocardiography (TEE) and/or fluoroscopic guidance. After the right internal jugular vein is accessed, a wire is advanced into the PA. Sequential dilation is performed and the ProtekDuo is advanced over the wire into position, while continuously monitoring its movement through the heart. This avoids cannulation of the groin permits early patient mobilization. Because it has the benefit of bypassing the RV with its drainage from the RA, the ProtekDuo decreases RV preload and decompresses the right side of the heart. The ProtekDuo has been used in conjunction with the TandemHeart (CardiacAssist, Pittsburgh, PA) pump or the CentriMag (Abbott, Pleasanton, CA) pump to provide temporary RV support and may provide blood flow of around 4.5 L/min (71). A centrifugal pump with cannulae positioned in the RA and PA can be used to provide both RV support and improved systemic oxygenation with the addition of an oxygenator to the circuit to create an oxy-RVAD (68, 69).

Several case reports and case series have described the use of the ProtekDuo for acute RHF in the setting of LVAD implantation, cardiogenic shock resulting from decompensated severe pHTN, and cardiogenic shock secondary to massive pulmonary embolism (72–74) (Table 3). Schmack and colleagues published a retrospective single center outcome analysis of all permanent LVAD recipients who required temporary RVAD using the ProtekDuo with TandemHeart (11 patients) (20). Ten patients (90.9%) were successfully weaned from temporary RVAD support, and 30-day survival was 72.7%; no severe RVAD associated complications were observed.

**TABLE 3:** Experience with percutaneous RVAD for the management of acute right heart failure.

References	RVAD device	Number of patients (Etiology of RHF)	Outcomes
Ravichandran et al. (15)	ProtekDuo RVAD	*N* = 17 patients 12 (post-LVAD implantation) 2 (post-heart transplant, one with early postoperative rejection and one with rejection several years after transplant) 2 (predominant RVF) 1 (biventricular failure)	● Deceased on temporary RVAD support: 41% ● Successfully weaned from temporary RVAD: 23% ○ Inability to wean device, required either surgical RVAD or durable RVAD: 35% ● Mean duration of RVAD support: 10.5 ± 6.5 days ● Complications in 35%: ○ 1 patient had epistaxis and hematemesis related to systemic anticoagulation ○ 1 patient had injury to left IJV (inability to advance catheter past RV due to tortuous anatomy) ○ 2 patients had intracranial bleeds on systemic anticoagulation ○ 2 patients had bleeding at catheter insertion site
Schmack et al. (20)	ProtekDuo RVAD	*N* = 11 patients (following LVAD implantation)	● 30-day survival: 72.2% ● Successfully weaned from temporary RVAD: 90.9% ○ 1 patient deceased on support ● Mean duration of RVAD support: 16.8 ± 9.5 days ● Mean ICU stay: 23.8 ± 16.5 days ● 3 patients (27.3%) died following multi-organ failure, 1 patient (9.1%) following intracranial bleed 12 days after RVAD explantation ● No temporary RVAD associated complications
Coromilas et al. (77)	ProtekDuo RVAD	*N* = 19 patients (following LVAD implantation)	● Comparison between perc-RVAD and surgical RVAD after durable LVAD implantation ● Hemodynamic parameters improved with perc-RVAD; these were sustained after device removal and similar to hemodynamic profiles of patients with surgical RVAD ○ CVP decreased: 15.9 ± 2.4–12.3 ± 3.2 mmHg, *P* < *0.001* ○ Cardiac index increased: 2.4 ± 0.5–3.5 ± 0.8 L/min/m^2^, *P* < *0.001* ● Patients with perc-RVAD required fewer blood transfusions and mechanically ventilated days compared to patients with surgical RVAD ● Among survivors, ICU and hospital days were fewer with perc-RVAD compared to surgical RVAD ○ ICU days: 21 (16–27) vs. 34 (27–46), *P* = *0.01* ○ Hospital days: 43.5 (30–66) vs. 91 (62–111) hospital days, *P* = *0.03* ● No significant difference in 30-day mortality with perc-RVAD compared to surgical RVAD (21.1% vs. 42.9%, *P* = *0.14*) ○ Trend toward higher rate of discharge free from hemodialysis (73.7% vs. 47.6%, *P* = *0.09*) in perc-RVAD patients
Badu et al. (3)	ProtekDuo RVAD	*N* = 40 patients 18 (postcardiotomy) 12 (cardiogenic shock) 10 (primary respiratory failure)	● In-hospital mortality: ○ Postcardiotomy: 11% ○ Cardiogenic shock: 58% ○ Respiratory failure: 40% ● Successfully weaned from temporary RVAD: ○ Postcardiotomy: 94% ○ Cardiogenic shock: 42% ○ Respiratory failure: 70% ● No temporary RVAD associated complications
Salna et al. (75)	ProtekDuo RVAD	*N* = 27 patients (following LVAD implantation)	● Successfully weaned from temporary RVAD: 86% ● Survival to discharge: 15% ● Median duration of RVAD support: 11 days ● Complications: new moderate-to-severe tricuspid regurgitation (36%), hemolysis (14%), cannula migration (7%) ● 3 patients (11%) required conversion to surgical RVAD ● Overall 1-year survival: 81%
Kremer et al. (19)	ProtekDuo RVAD	*N* = 10 patients (following acute MI)	● 30-day survival: 60% ● Successfully weaned from temporary RVAD: 40% ● Mean temporary RVAD time: 10.0 ± 7.4 days ● Significant reduction in CVP: 19.3 ± 2.7 vs. 8.2 ± 2.6 mm Hg, *P* < *0.001* ● Significant increase in SvO_2_: 52.8 ± 15.6 vs. 80.0 ± 6.0%, *P* < *0.001* ● Mean ICU stay: 18.6 ± 12.2 days ● 2 patients bridged to long-term paracorporeal RVAD ● Causes of death (*n* = 4): multiorgan failure, electromechanical dissociation, hemorrhagic stroke ● No temporary RVAD associated complications
Oliveros et al. (21)	ProtekDuo RVAD	*N* = 11 patients 4 (lung resection) 2 (acute respiratory distress) 1 (post-partum cardiomyopathy) 1 (post-valve surgery) 1 (following acute MI) 1 (post-LVAD) 1 (pulmonary embolism)	● 30-day survival: 82% ● 180-day survival: 72% ● Successfully weaned from RVAD: 54.5% ● Average length of RVAD support: 11–154 days ● Hospital length of stay: 12–223 days ● Complications: stroke (18.2%), sepsis (63.6%), massive GI bleed (45.5%), heparin-induced thrombocytopenia (54.5%)
Carrozzini et al. (76)	ProtekDuo RVAD	*N* = 3 patients (RV PGD after heart transplant)	● All patients weaned from temporary RVAD and successfully discharged without clinical or echocardiographic signs of RV dysfunction ● RVAD time: 4, 12, and 9 days ● Adverse events: AKI, IJV thrombosis, respiratory failure

In 2020, Kremer et al. described a series of 10 patients in which the ProtekDuo was used for temporary RV support after acute myocardial infarction and reported a 30-day survival rate of 60% (19). Oliveros and colleagues collected retrospective data on 11 consecutive patients who received a ProtekDuo for acute RHF over a 3-year period (21). The average length of support ranged from short-term (11 days) to long-term (154 days), and the main complications were stroke (18.2%), sepsis (63.3%), gastrointestinal bleed (45.5%), and heparin-induced thrombocytopenia (54.5%) (21).

A case series by Badu et al. reviewed 40 patients who received RVAD support with the ProtekDuo and compared outcomes among three subgroups based on the indications for RV MCS (postcardiotomy, cardiogenic shock, and primary respiratory failure) (3). In all, 94% of patients in the postcardiotomy group were weaned from RVAD support, 42% in the cardiogenic shock group, and 70% in the respiratory failure group. This compared favorably to the rate of weaning from surgical RVADs reported in the literature (49–59%). Furthermore, while published in-hospital mortality rates range from 42 to 50% for surgically placed RVADs and from 41 to 50% for pRVADs, mortality in this series was 11% in the postcardiotomy group, 58% in the cardiogenic shock group, and 40% in the respiratory failure group (3).

Salna and colleagues from Columbia University retrospectively reviewed their experience with the ProtekDuo in 27 patients who developed severe acute RHF following LVAD implantation (75). Implantation of the device was successful on the first day in all patients at a median of 1 day after LVAD implantation and the median duration of support was 11 days. Device weaning was successful in 86% of patients, with 15% in-hospital mortality. Major complications related to the device included new moderate-to-severe tricuspid regurgitation (36%), hemolysis (14%), and cannula migration (7%). Three patients (11%) required conversion to surgical RVAD. Overall survival to 1 year was 81% (75). A similar study by Ravichandran et al. reported on 17 patients who underwent insertion of a ProtekDuo for pRVAD, 12 of whom were post-LVAD implantation (15). The pRVAD was successfully weaned in 23% of patients without the need for home inotropes or urgent transplant due to RHF. In 35% of patients, the device could not be weaned and patients required either a surgical RVAD or durable RVAD. The remaining 41% of patients did not survive on RVAD support, which the authors concluded confirmed the poor prognosis of RHF (15).

The ProtekDuo has also been used in the setting of primary graft dysfunction (PGD) after heart transplant when the PGD was attributed to isolated RV failure. Carrozzini described the application of the ProtekDuo in 3 such cases (76). All patients had normal pulmonary artery pressures and PVR prior to transplantation. However, all patients required VA-ECMO support prior to transplant support due to end-stage biventricular failure. The diagnosis of RV PGD was concordant with the International Society for Heart and Lung Transplantation definition, based on signs of low cardiac output with increased central venous pressure, low pulmonary artery and capillary wedge pressures, low central venous oxygen saturation and reduced cardiac index, and echocardiographic findings demonstrating dilation and dysfunction of the RV with normal LV motion in the absence of cardiac tamponade. All patients underwent uneventful initiation of pRVAD and a maximal flow of 4 L/min was achieved in all cases. All patients were successfully weaned from RVAD support and discharged without clinical or echocardiographic signs of RV dysfunction (76).

Coromilas and colleagues compared patients who received pRVAD (19 patients) with those who received sRVAD (21 patients) after implantation of durable LVAD (77). pRVADs included patients who received an Impella RP or a ProtekDuo with either a TandemHeart or Centrimag pump. Patients with pRVAD required fewer blood transfusions and mechanically ventilated days compared to those with sRVAD. While there was no significant difference in 30-day mortality with the use of pRVAD compared with sRVAD, there was a trend toward a higher rate of discharge free from hemodialysis for patients who received a pRVAD. Additionally, among survivors, intensive care unit and hospital days were fewer with the use of pRVAD (77).

While experience with ProtekDuo cannula continues to grow, it is worth noting that this approach is relatively costly. Our group has moved to an alternative strategy that involves two access sites. We use a long 25 Fr venous cannula from the femoral vein for pump inflow and place a 19 Fr to 23 Fr cannula from the right internal jugular vein into the PA for return. This represents a slight modification from our most common cannulation strategy for venovenous ECMO (VV-ECMO), which uses right femoral vein and right internal jugular vein cannulation. We are comfortable adopting this approach because of our growing experience ambulating patients on VV-ECMO with a long venous cannula in place. Consequently, we do not view a long venous cannula as a contraindication to patient ambulation and participation in physical therapy.

## Conclusion

Acute RHF remains a major cause of global morbidity and mortality, irrespective of the injurious mechanism. Multiple studies have demonstrated poorer clinical outcomes in patients with RHF in the setting of left-sided heart failure, acute myocardial infarction, pHTN, and following major cardiac surgery. Historically, algorithms for the management of RHF focused on medical therapies to reverse the underlying cause, maintain adequate preload, reduce RV afterload, and enhance RV contractility. The development of MCS strategies to support the failing RV has been critically important to these sick patients, and in many instances life-saving. More recently, pRVAD allow patients to recover from RHF in the ICU setting without the necessity of returning to the operating room for additional procedures once RV function has improved. Even in their nascent use, these minimally invasive strategies have equivalent or better outcomes compared to sRVAD. Surgeons and interventionalists will continue to innovate and improve the quality and safety of devices used to support the failing RV, providing patients the best opportunity for survival.

## Author contributions

LJ: literature review and writing—original draft, review, and editing. DS: conceptualization and writing—review and editing. Both authors contributed to the article and approved the submitted version.
